# Time Course of COVID-19 Cases in Austria

**DOI:** 10.3390/ijerph17093270

**Published:** 2020-05-07

**Authors:** Hanns Moshammer, Michael Poteser, Kathrin Lemmerer, Peter Wallner, Hans-Peter Hutter

**Affiliations:** 1 Department of Environmental Health, Center for Public Health, Medical University Vienna, 1090 Vienna, Austria; hanns.moshammer@meduniwien.ac.at (H.M.); kathrin.lemmerer@meduniwien.ac.at (K.L.); peter.wallner@meduniwien.ac.at (P.W.); hans-peter.hutter@meduniwien.ac.at (H.-P.H.); 2 Karakalpak Medical University, Department of Hygiene, Nukus 230100, Uzbekistan; 3 International Society of Doctors for the Environment Austria, 1020 Vienna, Austria

**Keywords:** COVID-19, coronavirus, containment, public health

## Abstract

COVID-19 is an infectious disease caused by a novel coronavirus, which first appeared in China in late 2019, and reached pandemic distribution in early 2020. The first major outbreak in Europe occurred in Northern Italy where it spread to neighboring countries, notably to Austria, where skiing resorts served as a main transmission hub. Soon, the Austrian government introduced strict measures to curb the spread of the virus. Using publicly available data, we assessed the efficiency of the governmental measures. We assumed an average incubation period of one week and an average duration of infectivity of 10 days. One week after the introduction of strict measures, the increase in daily new cases was reversed, and the reproduction number dropped. The crude estimates tended to overestimate the reproduction rate in the early phase. Publicly available data provide a first estimate about the effectiveness of public health measures. However, more data are needed for an unbiased assessment.

## 1. Introduction

A novel Corona virus, SARS-CoV-2, was first observed in China in late 2019 [1]. That virus caused infections of the upper and lower airways in the form of an epidemic and soon pandemic disease [2] termed COVID-19. The virus first emerged in the Chinese city of Wuhan from where it first spread throughout China and then also reached neighboring countries in South-East Asia. Around the end of January 2020 first single cases were reported in the United States (23 January) and in Europe (France: 24 January; Germany: 27 January; Italy and United Kingdom: 31 January; and Spain: 1 February). These were initially single cases affecting either visitors from or to the original hot-spot areas in South-East China, or persons with direct contact to China-travelers. Accordingly, European countries resorted to a screening scheme mainly looking for suspects defined as symptomatic and either coming from a hot-spot area or having had close contact with a confirmed COVID-19 case. In the beginning, it was hoped that a wider spread could be prevented by the scheme and by a close survey of all contacts. In Europe, the virus first spread widely in Northern Italy (Lombardy, Veneto, recognized by the end of February), and it was rapidly distributed to neighboring countries like Austria. In Austria (as well as in Switzerland), the first case was reported on 25 February. In the weeks before, several Austrian suspects had been reported in the media but were always tested negative. In this scenario, Austrian authorities believed to have relatively good control over the spread of the virus. Later, we learned that skiing resorts already served as the first hub of transmission. It took several days until the authorities recognized the threat to public health and reacted accordingly. Besides enacting quarantine rules for the affected areas, the federal government of Austria also issued general laws restricting social contacts in all of Austria. This included the closure of schools, restaurants, many shops, cancellation of conferences, etc. Most of these restrictions went into force on Monday, 16 March. 

Daily reports on cumulative numbers of cases were issued through public media to support the state of public alert. Many important data, like the percentage of positive and negative test results, or demographic and medical details of the cases, were not reported publicly. Even anonymous data were not available for researchers. In the beginning, even daily numbers of performed PCR tests were not available but only cumulative numbers were reported. 

Here, we set out to analyze publicly available data on the time course of COVID-19 cases in Austria, to demonstrate the usefulness for public health and the limitations of this kind of data.

## 2. Materials and Methods

**Figure 1:**
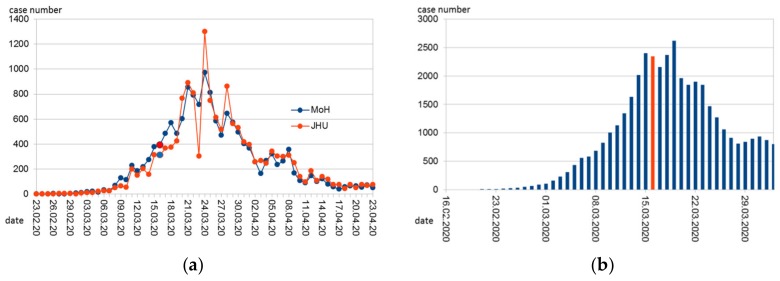
Time course of (**a**) daily reported COVID-19 cases in Austria according to the John Hopkins University (JHU, [3]) and the Austrian Ministry of Health (MoH, [5]); (**b**) Approximation of daily infections back-extrapolated from the reported cases and assuming 2 unreported cases per 1 reported. Marked in red (respectively in blue in the red series in Figure 1a is Monday, 6 March, 2020. On that day, most of the government-mandated measures to contain the epidemic began.

Data on newly reported cases from Austria on a daily basis were obtained from John Hopkins University (JHU) aggregated database [3]. This database has already been used for COVID-19 research by other researchers [4] and provides a comprehensive overview of newly diagnosed cases per day. When we first accessed the webpage of the Austrian Ministry of Health regarding COVID-19 information, the website only reported cumulative case numbers that were updated every day. In the meantime, the ministry also reports daily case numbers [5] with the date preferentially being reported as the date of the beginning of the disease, if known, otherwise (in that order) the date of the diagnosis or the date of reporting. The numbers from the Austrian database, therefore, display a smoother time-course than those from JHU. The Austrian data also seem to be one day earlier on average than the JHU data (Figure 1a). For the sake of consistency and because of the clearer definition (date of reporting only) we used the JHU data.

Assuming an incubation period of 7 days, one can back-extrapolate when the diagnosed cases were infected. This estimate is uncertain because the incubation period is not exactly 7 days, but between 5 and 10 days, and in individual cases possibly even more [6]. In addition, the incubation period indicates the time from infection to the appearance of symptoms. We do not know the time of the first symptoms, but the time of testing and reporting. It can be assumed that the time lag between the appearance of symptoms and the reporting has shortened with the better establishment of the reporting system. In spite of the mentioned shortcomings, we assumed a latency period between the first symptoms and positive testing and reporting of 2 days, and an incubation period that in 30% lasted for 5 days, in 35% for 6 days, in 20% for 7 days, in 10% for 8 days, and in 5% for 9 days, roughly mimicking a distribution of incubation periods with a median of about 6 days and for less than 5% of infected persons with an incubation period of 10 days or more [6]. This was deemed the best approximation available for describing the time course of infections.

The number of estimated infections per day, thus, only includes those persons that were later tested positive. The number of persons carrying the virus but never tested cannot be known for certain. A factor of 3 lies within the range of estimates: this was used by Czypionka and Reiss [7] and is in accordance with the results on a representative random sample of 1544 Austrians among which 5 were tested positive between April 1 and 6 [8]. Therefore, we assumed a latency of 2 days between the first symptoms and a positive test result, a distribution of the incubation period as described above, and a factor of 3 to estimate the true daily infections with SARS-CoV-2 in Austria.

Assuming that a person is infectious from day 2 to day 11 after infection (= day 0), one can estimate the number of potentially infectious people per day and can relate this to the number of people that were newly infected on that same day. This figure can be used to estimate how many people have been infected by an infectious person per day. Multiplied by 10, the total number of days that the person is infectious, the basic reproductive number (R_0_) can be estimated, or rather the effective reproduction number R_t_, which is a good approximation of R_0_ as long as the percentage of immune persons is small. This estimate is of course very uncertain. The percentage of unreported cases should not matter much as long as it remains constant over time as it affects both infectious and infected people, but a temporal trend in the percentage of unreported cases is not taken into account. The duration of the infectivity is not exactly 10 days and is subject to individual fluctuations [9]. A longer average duration of infectivity would increase the number of potentially infectious people per day. During steady state conditions, a doubling of the assumed period of infectivity would double the number of infectious people and thus halve the rate of newly infected by infectious persons. However, multiplying that rate by the number of days would again result in the same reproduction number. This inaccuracy should therefore not introduce a significant bias in the estimate of R_0_ as long as the number of daily infections is constant. In the initial phase, when the numbers increase steeply, this method would overestimate the reproduction number if the average duration of infectivity is incorrectly assumed and too long. Therefore, we also tried a model with 8 days of infectivity as a kind of sensitivity analysis.

## 3. Results

### 3.1. Time Course of Reports of New Cases and of Infections

The number of new cases (by date of reporting according to JHU) peaked on 26 March (Figure 1a). Due to its outstanding height, this peak is only roughly stated in the statistics of the JHU ("1.3k") and had to be calculated back due to the cumulative values provided for the current date (7 April 2020) as 1321. This high value is probably an artifact resulting from the late reporting of cases by newly recruited small laboratories.

The number of reported cases is also a function of the intensity of testing. It is understandable and known that the tests have been increased over time. Fluctuations over the course of the week in testing and reporting intensity are thus also conceivable.

It is obvious from the published figures that the government introduced strong measures at a time when the daily number of reported cases was still low and even lower than in mid-April when a relaxation of measures was already announced. Considering the much higher numbers of daily infections not yet documented, the measures were nevertheless timely. Extrapolating back to an approximation of the number of daily infections provides Figure 1b.

**Figure 2:**
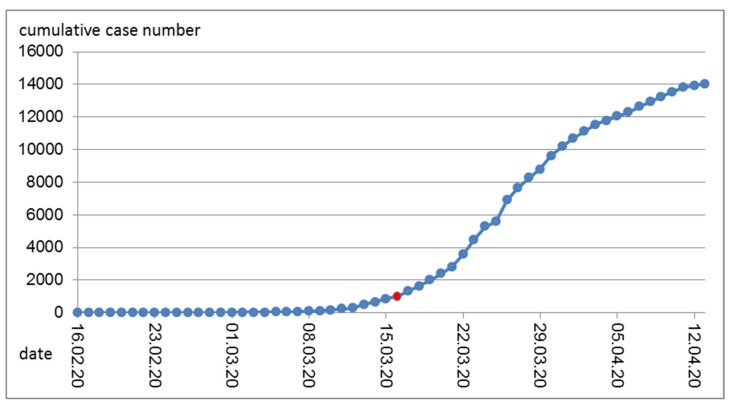
Cumulative COVID-19 cases in Austria over time. Marked in red is Monday, 16 March, 2020. On that day, most of the government-mandated measures to contain the epidemic began.

The cumulative number of cases is derived from the daily reports (Figure 2). Obviously, even with decreasing numbers of new cases, the cumulative number still increases. That number was mostly reported in public media and reached new heights every day and exceeded certain benchmarks (5000, 10,000) that each sparked a public outcry. 

### 3.2. Estimates of the Reproduction Rate R

**Figure 3:**
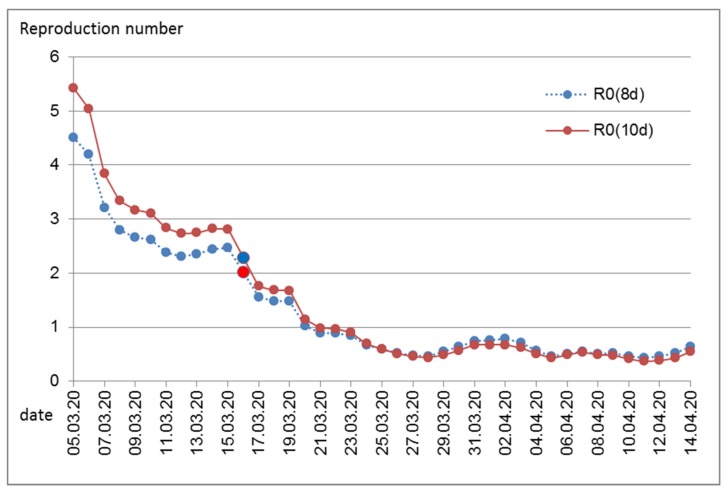
Estimated reproduction number per day. Marked in red is Monday, 16 March, 2020. On that day, most of the government-mandated measures to contain the epidemic began. Numbers calculated assuming an average duration of infectivity of 10 days, R_0_(10d), or of 8 days, R_0_(8d).

Assuming an average infectivity of 10 days, an average reproduction rate was estimated for each day (Figure 3). The high values during the early days are certainly artifacts of the poor data accuracy. This is especially true for the first cases that were tested and discovered when they had returned from a “risk area” and thus per definition could not be linked to an infectious source in Austria. In the beginning, borders were still open and infections occurred in skiing resorts, and infections were also caused by tourists that are not completely included among the Austrian confirmed cases. In these special settings and in hot-spot areas, local R_0_ values were certainly high. Although the total number of daily cases was low, the contact screening of these persons resulted in a relatively high percentage of total new cases. For contact screening, the timing of reporting does not accurately reflect the timing of symptom occurrence or the timing of infection.

Above all, the early values are probably an overestimation, as the tests only slowly reached a sufficient level at the beginning of the epidemic and therefore the number of unreported cases was higher at the beginning and the ratio between (later) infected and (older) infectious people was incorrectly estimated higher. In any case, it is clear that before the introduction of the measures, for around one week, a plateau between an R_0_ of 2 and 3 was reached, and with the introduction of the measures, the reproduction number has dropped to below 1.

## 4. Discussion

Governmental public health measures in Austria successfully curbed the transmission of the novel coronavirus. In accordance with our expectations, effects were visible after 1 week. This relatively short latency period between interventions and visibility of effects is important for further monitoring. A relaxation of strict measures is necessary both because of the economic and public health reasons. Enforced social distancing is also a threat to public health, including physical and mental health and social wellbeing [10]. A short latency period will enable the fine-tuning of restrictions, allowing both the necessary protection and the highest possible freedom and social contacts. 

For better prediction models, more data are necessary. There are certainly research needs in clinical medicine regarding therapeutic options for COVID-19 patients and regarding the development of vaccines. Likewise necessary in terms of individual but also public health interests are studies on individual predictors (genetics, life-style, and environment) of disease risk and prognosis [4,11,12,13]. Most important for public health is a better understanding and a more precise count of the percentage of undetected COVID-19 cases, including the size, determinants, and its change over time. Part of these questions could be answered when existing data are made publicly available for research; another part will need careful planning of dedicated studies in order to plan for future responses to COVID-19 activity. In an open letter to the Austrian Minister of Health on April 3rd, 2020, Austrian Scientists called for access to anonymous individual patient data [14]. To provide just one example, during the early phase, R_0_ can only be estimated based on individual data that include information on the whole infection chain.

Studies (serosurveys) involving antibody testing are now underway as a means to estimate the percentage of undiagnosed (and symptomless) infections. So far, these studies are planned in hot-spots (e.g., retirement homes) and groups of special interest. Population-based studies would be necessary to assess factors that predict timely access to testing. Such factors that should urgently be studied include age, socio-economic status, pre-existing diseases, ethnic minorities, and regions of living. 

The number of unreported cases also depends on the intensity of testing and has therefore decreased relatively over time. In the first phase of the Austrian epidemic, testing was restricted to “suspects” and a suspect was defined by (a) typical symptoms and (b) either contact with a known COVID-19 patient or a recent stay in a “risk area”. Originally, only South-East Asian countries were considered risk areas and later parts of Italy, before this definition was extended during the first weeks of March. At first, until the middle of March, only a few new cases were found that way. By definition, many inhabitants and visitors had been infected while abroad and therefore had no infectious contact within Austria. This would lead to an upward bias in the estimation of R_0_ from overall figures in the early phase. In that early phase, it was still hoped to contain the infection and each identified case triggered extensive in-depth contact testing. Therefore, the temporal course of case reporting in that phase did not reflect the temporal course of newly occurring cases. Only afterwards, and therefore concerning infections that had occurred since the beginning of March, was a general testing scheme established: every person perceiving the typical symptoms was urged to stay at home and call a hot-line. Health personnel would visit the patient to take nasal and pharyngeal swabs for PCR analysis. The patient was obliged to stay at home until tests confirmed them to be virus negative (or longer in the case of a positive test).

Therefore, starting around the first week of March, overall infection numbers are comparable over time to allow estimations of R_0_. Indeed, during that time (back-extrapolated to infection day 10 March), estimates of R_0_ are between 2 and 3, which is in accordance with international findings [15,16]. Only one week later, strict public health measures reduced R_0_ substantially. This effect was evident immediately (back-extrapolated to infection date 16 March, Figure 3), while the rate of daily infections still increased for several days (Figure 1b). The latter is not surprising considering the high number of recent infections and thus the high number of infectious persons during the early time of public health measures.

Persons might remain infectious for more than 10 days [9]. Information on the duration of infectivity comes mostly from cases with severe infection, mostly from hospitalized patients. The majority of infected persons will have experienced no or only slight symptoms. In that case, a shorter duration of infectivity is to be expected. Cases with severe symptoms are likely to reduce social contacts or will be hospitalized where social contacts are reduced through hospital hygiene measures. Therefore, we also assumed an average duration of infectivity of 8 days only. As expected, this resulted in somewhat lower reproduction numbers during the early time of the epidemic in Austria. Generally, it seems that assumptions regarding the average duration of infectivity are not very influential in the estimation of the reproduction number.

The development of R_0_ over time shows that the reproduction number was substantially declining, even before immunity could have been protecting larger parts of the population. At the time when the measures including the closure of schools, restaurants, and shops and tight regulations on personal mobility and contacts where announced, many skeptical comments about their necessity were voiced. Maybe because of the political shock triggered by the poorly managed outbreak in a skiing resort in Tyrol that also induced international critique, Austrian politicians acted timely and strong. While the effectiveness of these measures now stands, without doubt, the necessity of at least some of the measures remains open to discussion. Unfortunately, we lack a second world to run a controlled trial. Different countries have introduced different public health measures with different rigor and different timing, but countries also differ by demographic indices and by social norms and political culture. Chance events affect the timing and spread of diseases. Therefore, conclusive comparisons between different countries are challenging when it comes to the evaluation of specific measures. 

We acknowledge that our models are quite simple as they were primarily meant for educational purposes. They do not provide information on uncertainties or confidence intervals. On top of statistical uncertainties, uncertainties due to the limitations of the reported and publicly available data must also be considered.

## 5. Conclusions

Public health measures in Austria were successful. Infection rates reached low levels within a period comparable in duration to that in China. The public health measures also had beneficial (e.g., improved air quality) and adverse side effects (e.g., social isolation, anxiety, and lack of physical activity).

Currently (end of April), a gradual termination of the shutdown is being considered in the Austrian political arena. It must be emphasized that alleviating the containment policy will need constant monitoring and fine-tuning over the upcoming months. This will be best achieved with a maximum of (anonymous) data available both from existing databases and from new and on-going studies. 

Free access to the necessary data for research and public health management is vital for optimal management of public health measures.
